# Exosomes derived from adipose-derived stem cells overexpressing glyoxalase-1 protect endothelial cells and enhance angiogenesis in type 2 diabetic mice with limb ischemia

**DOI:** 10.1186/s13287-021-02475-7

**Published:** 2021-07-15

**Authors:** Xing Zhang, Yihong Jiang, Qun Huang, Zhaoyu Wu, Hongji Pu, Zhijue Xu, Bo Li, Xinwu Lu, Xinrui Yang, Jinbao Qin, Zhiyou Peng

**Affiliations:** Department of Vascular Surgery, Shanghai Ninth People’s Hospital, Shanghai Jiaotong University School of Medicine, Shanghai, 200011 China

**Keywords:** Glyoxalase-1, Adipose-derived stem cells, Exosomes, T2DM, Endothelial cells, Angiogenesis

## Abstract

**Background:**

Diabetic limb ischemia is a clinical syndrome and refractory to therapy. Our previous study demonstrated that adipose-derived stem cells (ADSCs) overexpressing glyoxalase-1 (GLO-1) promoted the regeneration of ischemic lower limbs in diabetic mice, but low survival rate, difficulty in differentiation, and tumorigenicity of the transplanted cells restricted its application. Recent studies have found that exosomes secreted by the ADSCs have the advantages of containing parental beneficial factors and exhibiting non-immunogenic, non-tumorigenic, and strong stable characteristics.

**Methods:**

ADSCs overexpressing GLO-1 (G-ADSCs) were established using lentivirus transfection, and exosomes secreted from ADSCs (G-ADSC-Exos) were isolated and characterized to coculture with human umbilical vein endothelial cells (HUVECs). Proliferation, apoptosis, migration, and tube formation of the HUVECs were detected under high-glucose conditions. The G-ADSC-Exos were injected into ischemic hindlimb muscles of type 2 diabetes mellitus (T2DM) mice, and the laser Doppler perfusion index, Masson’s staining, immunofluorescence, and immunohistochemistry assays were adopted to assess the treatment efficiency. Moreover, the underlying regulatory mechanisms of the G-ADSC-Exos on the proliferation, migration, angiogenesis, and apoptosis of the HUVECs were explored.

**Results:**

The G-ADSC-Exos enhanced the proliferation, migration, tube formation, and anti-apoptosis of the HUVECs in vitro under high-glucose conditions. After in vivo transplantation, the G-ADSC-Exo group showed significantly higher laser Doppler perfusion index, better muscle structural integrity, and higher microvessel’s density than the ADSC-Exo and control groups by Masson’s staining and immunofluorescence assays. The underlying mechanisms by which the G-ADSC-Exos protected endothelial cells both in vitro and in vivo might be via the activation of eNOS/AKT/ERK/P-38 signaling pathways, inhibition of AP-1/ROS/NLRP3/ASC/Caspase-1/IL-1β, as well as the increased secretion of VEGF, IGF-1, and FGF.

**Conclusion:**

Exosomes derived from adipose-derived stem cells overexpressing GLO-1 protected the endothelial cells and promoted the angiogenesis in type 2 diabetic mice with limb ischemia, which will be a promising clinical treatment in diabetic lower limb ischemia.

**Supplementary Information:**

The online version contains supplementary material available at 10.1186/s13287-021-02475-7.

## Background

Diabetes mellitus (DM) is a chronic health condition and associated with several comorbidities. The probability of diabetic patients suffering from ischemic lower extremity disease is 2–4 times that of non-diabetic patients, and the risk of amputation is 4–5 times that of normal people [1]. Diabetic lower limb ischemia has a high incidence, insidious onset, and long and protracted course. It often leads to non-healing diabetic foot ulcers and ischemic necrosis of the lower extremities which result in minor and major amputation, multiple organ dysfunction syndrome, and death [2–4]. Conventional therapeutic approaches in clinical practice such as drug therapy, by-pass open surgery, and percutaneous transluminal angioplasty have resulted in an unsatisfying long-term outcome [5].

Over the past decade, the rapid development of regenerative medicine has made the adipose-derived stem cells (ADSCs) become one of the most promising stem cells to use in lower extremity ischemia due to their abundant sources, high enrichment property, high proliferation rate, and low immunogenicity. Several research studies have proved that the ADSCs promote skin wound healing and ischemic tissue angiogenesis in animal models [6, 7]. However, advanced clinical prospective studies have shown an unsatisfying long-term clinical effect of autologous transplantation of the ADSCs [8, 9]. Studies have revealed that autologous transplanted ADSCs have limited survivability in the unfavorable environment around the target damaged vessels [10]. Additionally, stem cells can hardly differentiate into specific types of cells in vivo to replace the cells at the site of injuries [11]. Further, ADSCs transplantation has safety risks such as microvascular embolism and tumor formation in vivo.

Lately, many researchers have discovered that ADSCs play a role in promoting tissue repair mainly through paracrine function in vivo [12]. Among the many substances secreted by the ADSCs, exosomes (Exos) have become a research hotspot of “cell-free” treatment strategies in the field of stem cell therapy in recent years. Adipose-derived stem cell exosomes (ADSC-Exos) are membranous microvesicles with a size of 40–160 nm (100 nm on average), which can be fused out of the cell through the membrane and then taken up by the target cells through endocytosis. The ADSC-Exos mediate positive biological therapeutic effects through their contents of growth factors, cytokines, proteins, lipids, microRNA, and mRNA, similar to the parent ADSCs. The ADSC-Exos have no immunogenic and tumorigenic properties and is highly stable and can be transported for long distances through the biofluids. These cell-free vesicles can be used to replace stem cells in transplantation therapies [13]. Animal experiments have shown that autologous transplanted ADSC-Exos can resist apoptosis of target cells, promote proliferation, migration, and tube formation of endothelial cells, and promote angiogenesis in ischemic tissues, but the specific underlying mechanism is still unclear [14]. Therefore, the auto-transplantation of ADSC-Exo has good clinical application potential in diabetic patients with lower limb ischemia.

Studies have shown that the continuous high-glucose environment in diabetic patients may destroy endothelial cells and inhibit angiogenesis through advanced glycosylation end products, pro-inflammatory microenvironment, and induction of oxidative stress mechanisms [15, 16]. As the key rate-limiting enzyme in the glyoxalase system, GLO-1 catalyzes the transfer and isomerization of methylglyoxal produced in glycolysis into S-D-lactoylglutathione and then into lactic acid to reduce the excessive accumulation of toxic end products caused by the oxidative stress in cells. Studies have confirmed that the overexpression of the GLO-1 downregulated the ROS inside the vascular endothelial cells and protected their functions [17]. The overexpression of GLO-1 in bone marrow stem cells increased their angiogenic ability [18]. Our previous research also proved that the overexpression of the GLO-1 ameliorated the proangiogenic ability of ADSCs in diabetic mice with hindlimb ischemia [19]. But the GLO-1-overexpressing-ADSCs (G-ADSCs) exhibited an extremely low survival rate after transplantation in vivo and the ethical issues related to the lentiviral transfection restricted its clinical application [20].

Therefore, the objectives of the current study were to acquire the G-ADSCs secreted exosomes (G-ADSC-Exos); elucidate the effect of the G-ADSC-Exos on the proliferation, migration, and tube formation of endothelial cells under high glucose; and transplant the G-ADSC-Exos into the ischemic lower limbs of type 2 diabetic (T2DM) mice to examine its therapeutic efficiency in vivo. Furthermore, this study aimed to explore the changes in the related signaling pathways and paracrine factors to gain new ideas and theoretical bases for the clinical treatment of diabetic lower limb ischemia.

## Methods

### Animals

Four-week-old wild-type (WT) C57/BL mice and 8-week-old T2DM mice C57BL/KsJ-db/db (db/db) were purchased from Shanghai Research Center for Model Organisms (Shanghai, China) and reared specific pathogen-free. All the in vivo animal experiments were approved by the Animal Ethics Committee of Shanghai Ninth People’s Hospital, Shanghai Jiao Tong University School of Medicine.

### Isolation, culture, and characterization of ADSCs

The subcutaneous adipose tissue was acquired sterile from the areas of groin and armpits of 4-week-old male WT C57/BL6 mice, digested using NB4 collagenase (Nordmark, Uetersen, Germany), shaken to form a homogenous mixture, and seeded on culture plates with Dulbecco’s modified Eagle medium (DMEM) containing normal glucose (5.5 mmol/L), 10% fetal bovine serum (FBS), 100 U/mL penicillin, and 100 mg/mL streptomycin for primary culture. The cultures were incubated at 37 °C under 5% CO_2_. The ADSCs of the third passage were collected, washed with phosphate-buffered saline (PBS), and incubated with anti-mouse antibodies of CD29, CD90, CD45, and CD34 for 25 min at 4 °C in the dark. The isotype antibodies were used as negative controls. Flow cytometry (Beckman Coulter, Fullerton, CA, USA) was performed on the cells after washing 3 times with PBS as described previously [21].

### Glyoxalase-1 overexpression in the ADSCs

To stably obtain large numbers of ADSC-derived exosomes, lentiviral vectors were used for gene transfection of ADSCs. The lentiviral vectors containing *GLO-1* and *GFP* (green fluorescent protein) gene were purchased from Hanbio Biotechnology (Shanghai, China). The lentivirus was incubated with the ADSCs for 24 h at a multiplicity of infection (MOI) of 30. The expression of GFP in the ADSCs was observed using a fluorescence microscope (Olympus IX81, Tokyo, Japan, http://www.olympus-ims.com) to identify the transfection efficiency. The expression of the GLO-1 in the G-ADSCs was confirmed at the levels of protein and mRNA by Western blot and q-PCR, respectively.

### Isolation and characterization of the G-ADSC-Exos

After the successful production of the ADSCs stably overexpressing the GLO-1, the G-ADSC-derived exosomes (G-ADSC-Exos) were isolated as previously described [22]. First, the cell samples were centrifuged at 300×*g* for 10 min and the supernatant was collected. Then, the garnered supernatant was centrifuged at 2000×*g* for 10 min and the supernatant was collected. Finally, the garnered supernatant was centrifuged at 10,000×*g* for 3 h and the supernatant was discarded while saving the pellet. After resuspension of the precipitate with PBS, nanoparticle tracking analysis (NTA) (ZetaView, Particle Metrix, Meerbusch, Germany) was performed to measure the diameter of the exosomes. The morphological characteristics of the exosomes were observed using a transmission electron microscope (TEM) (JEM-2100F, Japan Electronics, Tokyo, Japan). The exosomal markers such as CD9, CD63, CD81, and TSG101 and the expression of the GLO-1 in the G-ADSC-Exos were looked at using the Western blot.

### Coculture of HUVECs and G-ADSC-Exos under high-glucose environment

Human umbilical vein endothelial cells (HUVECs) were purchased from the Shanghai Cell Resource Center at the Institute of Life Sciences (Shanghai, China) and cultured with high-glucose (33.3 mmol/l) DMEM with 5% fetal bovine serum (Gibco, Waltham, MA, USA) and 1% antibiotic/antimycotic solution (Gibco) as previously described [23]. The HUVECs were then cocultured with CM-Dil labeled G-ADSC-Exos at 37 °C under 5% CO_2_ for 24 h. The G-ADSC-Exos engulfed by the HUVECs were observed using a fluorescence microscope (Olympus IX81, Tokyo, Japan, http://www.olympus-ims.com). The DAPI was used to stain nuclei, and phalloidin was used to stain the cytoskeletons of the HUVECs.

### Cell proliferation and apoptosis assay of HUVECs

The cell counting kit-8 (CCK-8; Abcam, Cambridge, UK, https://www.abcam.cn) was used to identify the most effective therapeutic concentration of the G-ADSC-Exos on the HUVECs. The HUVECs were seeded on 96-well plates at a density of 2 × 10^3^ cells/well in high-glucose conditions and cocultured with PBS, 25 μg/mL ADSC-Exos, 50 μg/mL ADSC-Exos, 100 μg/mL ADSC-Exos, 25 μg/mL G-ADSC-Exos, 50 μg/mL G-ADSC-Exos, and 100 μg/mL G-ADSC-Exos, respectively. The CCK-8 solution was added to the wells at 10 μL/well at 0, 24, 48, and 72 h, after the incubation for 2 h at 37 °C. A microplate spectrophotometer (Varioskan; Thermo Fisher Scientific, Eugene, OR, USA) was employed to measure the optical density (OD) at 450 nm wavelength.

For apoptosis assay, the HUVECs were cultured in high-glucose DMEM for 48 h adding PBS, 100 μg/mL ADSC-Exos, and 100 μg/mL G-ADSC-Exos, respectively. The Annexin V PE/7-AAD apoptosis detection kit (Solarbio Science & Technology Co. Ltd., Beijing, China) and flow cytometry (Beckman Coulter, Fullerton, CA, USA) were used to measure the apoptosis ratio as previously mentioned [24]. Finally, the Western blot assay was performed on the lysate of the HUVECs cocultured with PBS, ADSC-Exos, and G-ADSC-Exos using antibodies against Caspase-3, Bcl-2, Bax, and β-actin (1:500; Abcam, Cambridge, UK).

### Wound-healing, transwell migration, and tube formation assay of the HUVECs

To elucidate how the G-ADSC-Exos influenced the migration and angiogenic ability of the HUVECs under high-glucose conditions during the process of wound-healing, transwell migration, and tube formation assay were performed as previously described [21]. For the wound-healing assay, after scraping and washing with PBS, the HUVECs and 100 μg/mL G-ADSC-Exos were co-incubated under a high-glucose environment for 24 h, and then the coculture was viewed using an inverted microscope (Olympus, Tokyo, Japan, http://www.olympus-ims.com) at 0 and 24 h. The Image J software (National Institutes of Health, Bethesda, MD, USA, https://imagej.nih.gov/ij/) was used to measure the area of the gaps.

The transwell migration assay was performed with a Boyden chamber and a polyethylene terephthalate (PET) membrane (R&D Systems Inc., Minneapolis, MN, USA). One hundred micrograms per milliliter G-ADSC-Exos and 2 × 10^3^ HUVECs were added onto the upper chamber containing 100 μL high-glucose DMEM with 0.5% FBS, and a 500-μL high-glucose DMEM was added to the lower chamber. After 24 h, a cotton swab was used to wipe the Matrigel and cells from the upper chamber, 4% paraformaldehyde was used to fix the HUVECs migrated through the PET membrane, and then 1% crystal violet in 2% ethanol was used to stain the fixed HUVECs. An inverted microscope (Olympus, Tokyo, Japan, http://www.olympus-ims.com) was adopted to capture the images.

For the tube formation assay, the HUVECs were cocultured with 100 μg/mL G-ADSC-Exos in high-glucose DMEM for 48 h, then seeded on Matrigel in a culture dish and incubated at 37 °C for 12 h. An inverted microscope (Olympus, Tokyo, Japan http://www.olympus-ims.com) was adopted to capture the images, and the ImageJ software (National Institutes of Health, Bethesda, USA, https://imagej.nih.gov) was utilized to calculate the cumulative tubular growth. All the experiments mentioned above were performed three times.

### Construction of T2DM mouse limb ischemia model and treatment of limb ischemia

Twenty-four male 8-week-old T2DM C57BL/KsJ-db/db mice (Shanghai Research Center for Model Organism, China) were anesthetized with the intraperitoneal injection of 0.3 ml/kg of 1% chloral hydrate. The skin of the left hindlimb was shaved, disinfected with iodophor, and the sterile drapes were placed. A longitudinal incision of 5 mm from the groin to the inner thigh was made, the membranous vascular sheath was gently pierced to expose and separate the femoral artery, vein, and nerve under a 20-fold Olympus SZ61 stereoscopic microscope (Olympus, Tokyo, Japan, http://www.olympus-ims.com). The femoral artery was ligated at the distal end of the common femoral artery and the proximal end of the superficial femoral artery with 7–0 surgical sutures, respectively. This model by a simple low ligation of the femoral artery was reported to mostly mimic clinical peripheral vascular disease, and suitable for studying the regeneration of blood vessels and striated muscle in the field of regenerative medicine [25]. The 24 mice were randomly divided: the ADSC-Exo group (*n* = 8), the G-ADSC-Exo group (*n* = 8), and the PBS control group (*n* = 8). After 24 h, the sites of the gastrocnemius, gracilis, and quadriceps muscles in the three groups received injections of 2 mL PBS with 100 μg/mL ADSC-Exos, 2 mL PBS with 100 μg/mL G-ADSC-Exos, and 2 mL PBS, respectively. The blood flow was evaluated with a laser Doppler perfusion imager (moorFLPI; Moor Instruments, Devon, UK) noninvasively on the first, seventh, and 28th day after implantation. The blood perfusion ratio of the ischemic limbs compared to the contralateral limbs was calculated.

### Histological analysis

The mice from the three groups were anesthetized and perfusion-fixed on day 28. The hindlimb muscles were harvested and the Masson’s trichrome staining was applied to evaluate the structural integrity of the ischemic muscles. The immunohistochemical and immunofluorescence staining were adopted to observe the density of microvessels as described previously [21]. For the immunofluorescence staining of the muscle sections, the α-smooth muscle actin (α-SMA) was stained with FITC (Abcam), and the nucleus was stained with DAPI (Dako).

### Detection of paracrine factors in the G-ADSC-Exos

The protein expression of tumor necrosis factor-α (TNF-α), vascular endothelial growth factor (VEGF), insulin-like growth factor-1 (IGF-1), hepatocyte growth factor (HGF), platelet-derived growth factor (PDGF), epidermal growth factor (EGF), and fibroblast growth factor (FGF) in the ADSC-Exos and G-ADSC-Exos were measured using ELISA kits (R&D Systems Inc., Minneapolis, MN, USA) in vitro. All the experiments mentioned above were performed three times.

### Detection of ROS production in the HUVECs

After cocultured under high-glucose conditions with PBS, 100 μg/mL ADSC-Exos, and 100 μg/mL G-ADSC-Exos for 48 h, respectively, the HUVECs were labeled with 2, 7-dichlorofluorescein diacetic acid (DCFH-DA), and the level of intracellular ROS (reactive oxygen species) was detected with a ROS analysis kit (Beyotime, Shanghai, China). Briefly, the adherent HUVECs were incubated with the DCFH-DA at a final concentration of 5 mM at 37 °C for 20 min and then washed three times with PBS. An inverted fluorescence microscope (Olympus IX81, Tokyo, Japan, http://www.olympus-ims.com) was used to analyze the production level of ROS in the HUVECs immediately.

### Detection of signaling pathways in vitro and in vivo

For the in vitro experiment, total protein was isolated from the HUVECs cocultured with PBS, 100 μg/mL ADSC-Exos and 100 μg/mL G-ADSC-Exos under high-glucose conditions for 48 h, respectively. And for in vivo experiment, total protein was isolated from the muscle tissues of the ADSC-Exo group, the G-ADSC-Exo group, and the PBS group, respectively, and the proteins were probed with the following antibodies: anti-eNOS and p-eNOS antibodies, anti-AKT and p-AKT antibodies, anti-ERK antibody, anti-P-38 and p-P-38 antibodies, and anti-AP-1, anti-ASC, anti-Caspase-1, anti-NLRP3, anti-IL-1-β, and anti-β-actin antibodies (1:500; Abcam). Signals were detected after incubating with an HRP-labeled secondary antibody and a chemiluminescent substance (Roche, Basel, Switzerland), and then the images were collected with a LAS3000 machine (GE Healthcare Life Sciences, Pittsburgh, USA).

### Statistical analysis

The mean ± standard deviation was used to describe parametric values. The one-way analysis of variance and two-tailed Student’s *t*-test were performed to compare data between groups using GraphPad Prism version 6.0 (GraphPad, La Jolla, CA, USA, http://www.graphpad.com) and SPSS version 25.0 (IBM-SPSS Inc., Armonk, NY, USA, https://www.ibm.com). A *p* < 0.05 was defined as statistically significant.

## Results

### Establishment of ADSCs stably overexpressing GLO-1

**Fig. 1:**
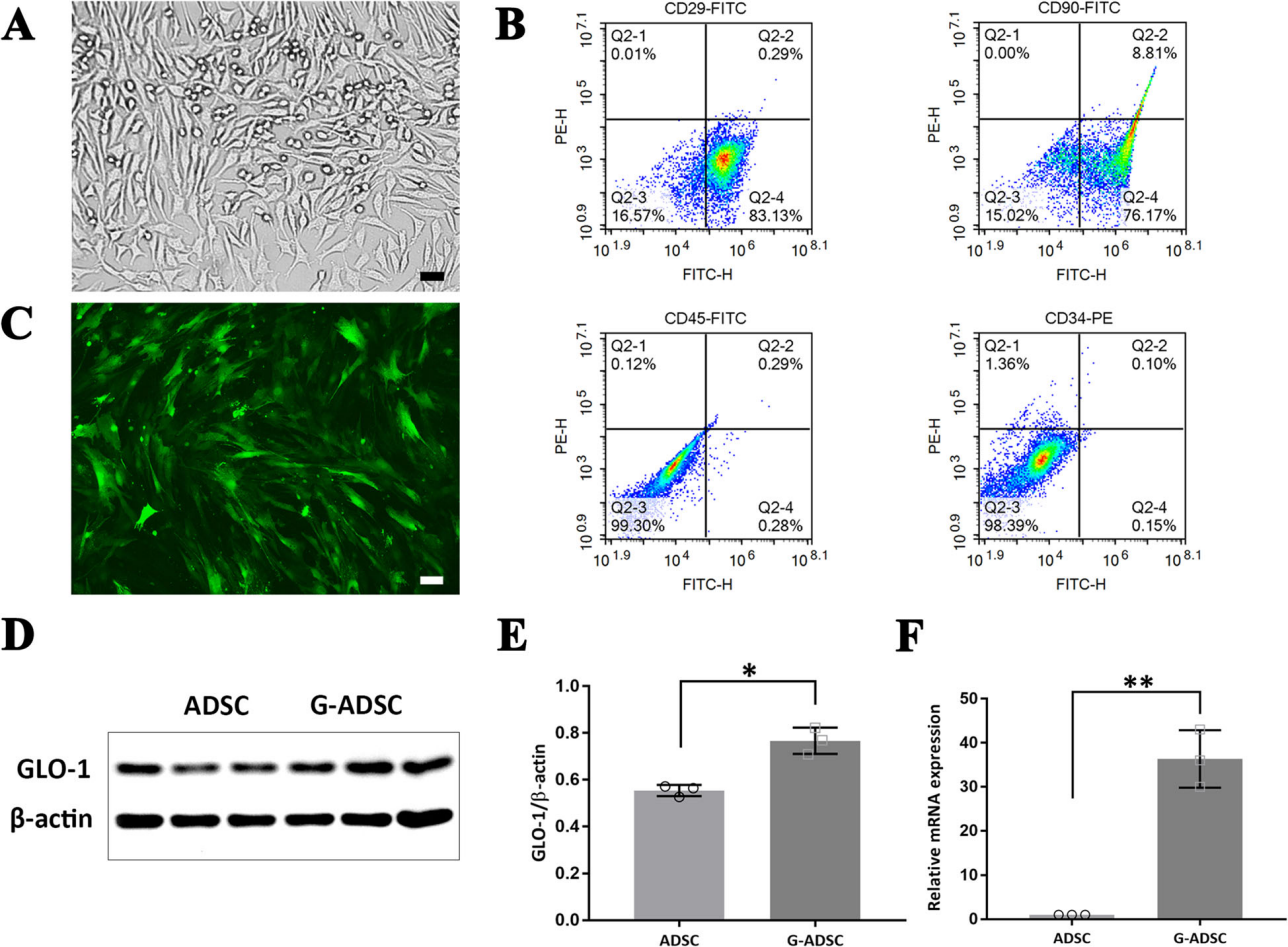
Characterization of the ADSCs and the Glyoxalase-1 overexpression in ADSCs. P3-ADSCs in vitro were spindle-shaped and fibroblast-like under the microscope (**A**). Identification of the ADSC surface markers using flow cytometry showed that the cells were positive for CD29 and CD90, and negative for CD45 and CD34 (**B**). The G-ADSCs emitted green fluorescence under a fluorescence microscope (**C**). Western blot (**D**, **E**) and q-PCR (**F**) analyses proved the successful overexpression of the GLO-1 in the G-ADSCs. All experiments above were repeated three times (*n* = 3). A two-tailed Student’s *t*-test was performed to compare data between groups. *P < 0.05; **P < 0.01; scale bar = 100 μm. P3, passage 3; G-ADSCs, GLO-1-overexpressing adipose-derived stem cells

The cells passaged to the third generation in vitro were spindle-shaped and fibroblast-like under the microscope. Identification of the ADSC surface markers using flow cytometry showed that cells were positive for CD29 (83.1% ± 2.7%) and CD90 (76.2% ± 3.3%), and negative for CD45 (0.3% ± 0.1%) and CD34 (0.2%±0.1%), which was consistent with the previously reported surface marker characteristics of the ADSCs (Fig. 1A, B) [21]. Since the isolation and collection of a large number of exosomes from the G-ADSCs required the stable overexpression of the GLO-1 in passaged and amplified G-ADSCs, we chose the lentivirus transfection method as previously described [19]. The emission from the green fluorescence G-ADSCs was observed employing fluorescence microscopy (Fig. 1C), Western blot (Fig. 1D, E), and q-PCR (Fig. 1F), and the respective analyses together proved the successful overexpression of the GLO-1 in the G-ADSCs.

### Isolation and characterization of the G-ADSC-Exos

**Fig. 2:**
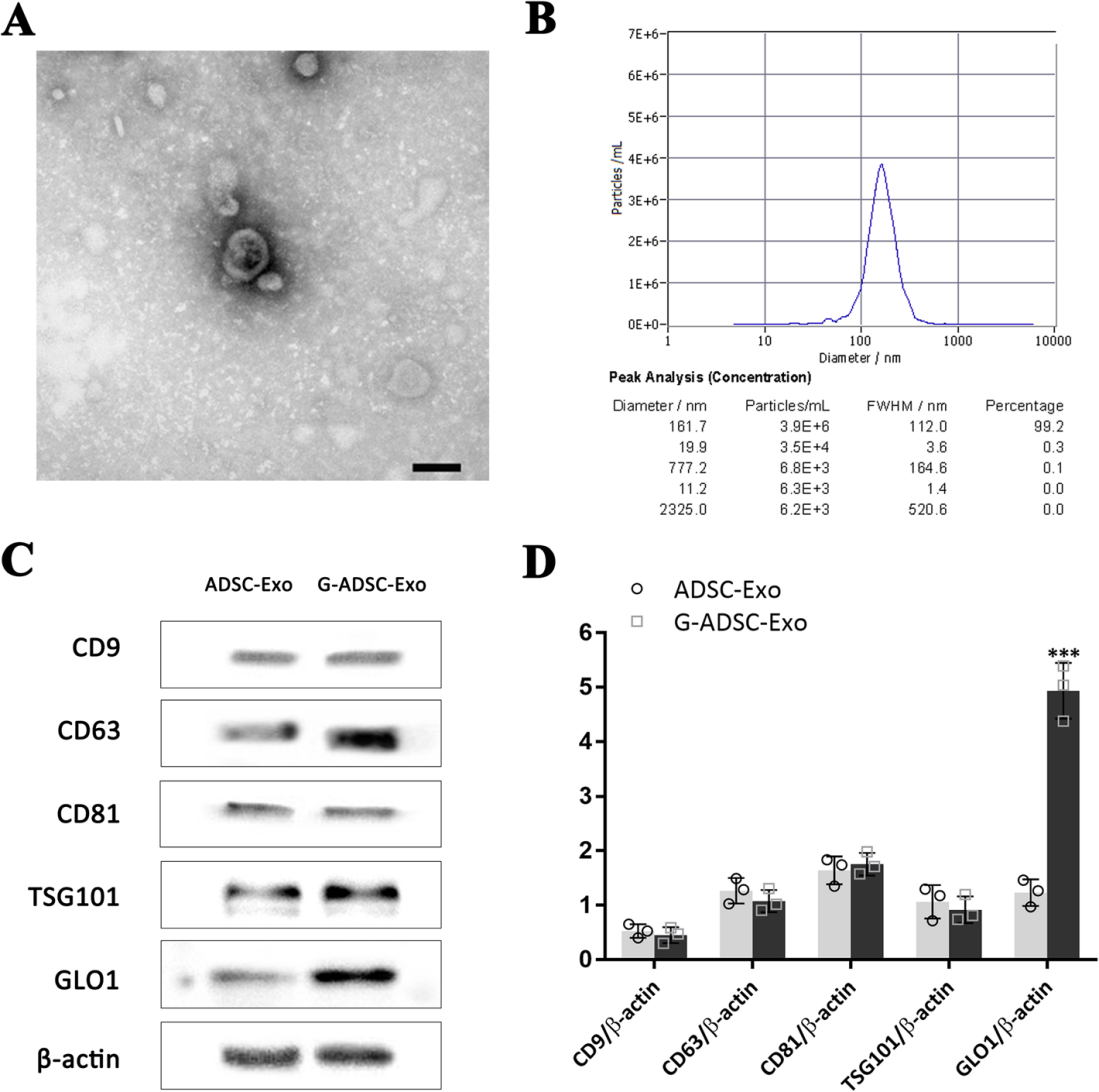
Characterization of the G-ADSC-Exos. The exosomal membrane exhibited a saucer-like structure under the transmission electron microscope (**A**). The NTA analysis demonstrated that 99.2% of exosomes were 161.7 nm in diameter (**B**). Western blot (**C**) and statistical analysis (**D**) showed that both ADSC-Exos and G-ADSC-Exos expressed exosomal surface markers of CD9, CD63, CD81, and TSG101, and the G-ADSC-Exo expressed a significantly higher level of GLO-1. All experiments above were repeated three times (*n* = 3). A two-tailed Student’s *t*-test was performed to compare data between groups. Scale bar = 100 nm. ***P < 0.001. G-ADSC-Exos, G-ADSC-derived exosomes

Exosomes derived from the ADSCs and G-ADSCs were successfully isolated with the exosomal membrane exhibiting a saucer-like structure under the transmission electron microscope (Fig. 2A). The NTA analysis demonstrated that 99.2% of exosomes were 161.7 nm in diameter (Fig. 2B). The Western blot and statistical analysis showed that the both ADSC-Exos and G-ADSC-Exos expressed exosomal surface markers of CD9, CD63, CD81, and TSG101, while G-ADSC-Exos expressed a significantly higher level of the GLO-1 (*P* < 0.001), which indicated the establishment of bio-modified exosomes with the high abundance of the GLO-1 protein (Fig. 2C, D).

### The G-ADSC-Exos ameliorated the viability and inhibited the apoptosis of HUVECs under high-glucose conditions

**Fig. 3:**
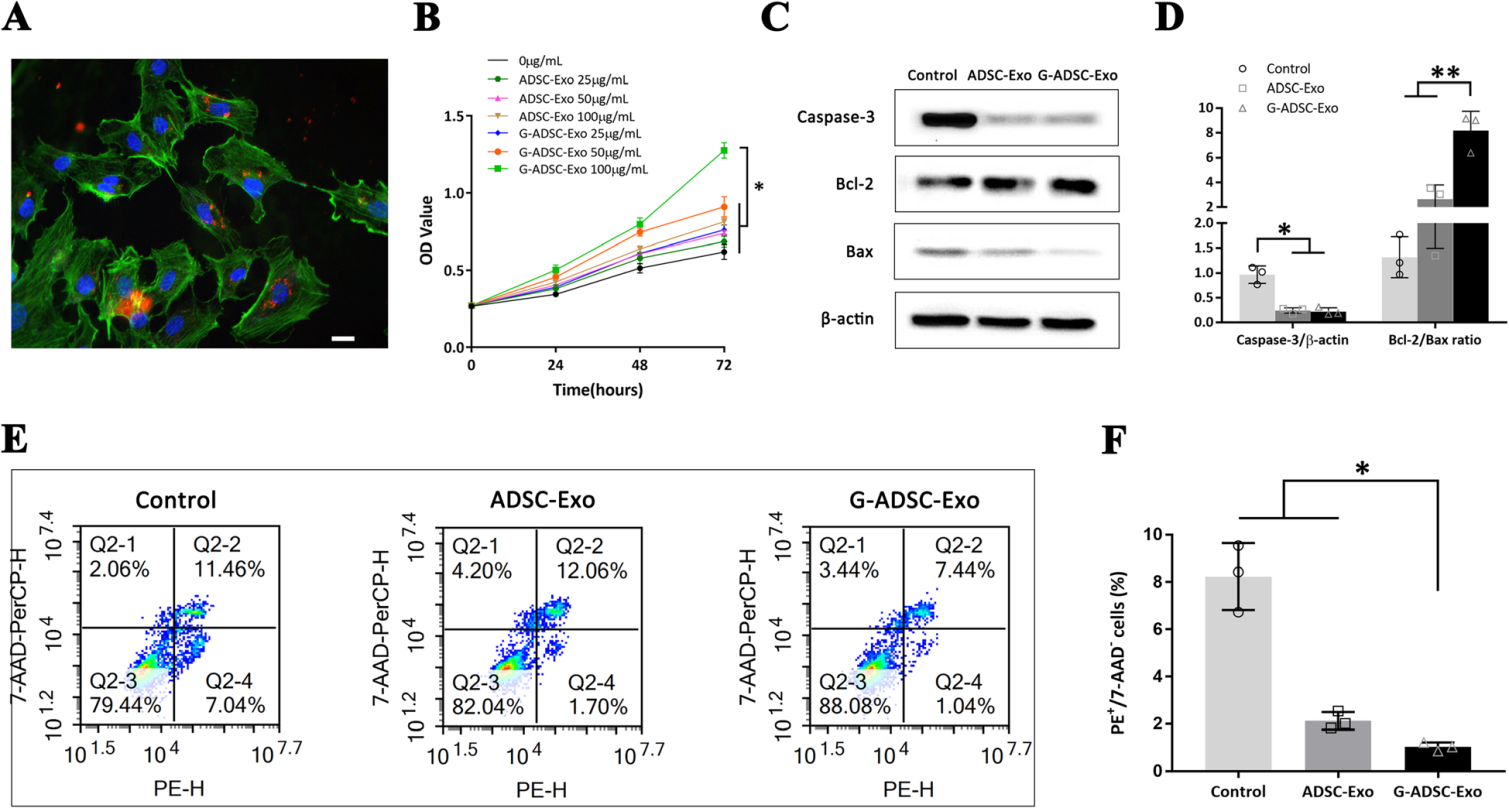
Cell proliferation and apoptosis assay of the HUVECs. The CM-Dil labeled G-ADSC-Exos contained red particles localized around the blue-stained nucleus within the green-stained cytoskeleton of the HUVECs under the fluorescence microscope (**A**). The CCK-8 analysis indicated that the most effective therapeutic concentration of the G-ADSC-Exos was 100 μg/mL (**B**). The G-ADSC-Exo group showed a significantly higher Bcl-2/Bax ratio than both ADSC-Exo and control groups (**C**, **D**). The G-ADSC-Exo group showed a significantly lower apoptotic percentage than both ADSC-Exo and control groups by flow cytometry analysis with a sample size of 10,000 cells with 3 biological replicates (**E**, **F**). All experiments above were repeated three times (*n* = 3). The one-way analysis of variance was performed to compare data between groups. Scale bar = 20 μm; *P < 0.05; **P < 0.01. G-ADSC-Exos, G-ADSC-derived exosomes

After co-incubation for 24 h, the CM-Dil labeled G-ADSC-Exos were red particles localized around the blue-stained nucleus within the green-stained cytoskeleton of the HUVECs under a fluorescence microscope, which proved the engulfment of the G-ADSC-Exos by the HUVECs (Fig. 3A). The CCK-8 analysis demonstrated that at 72 h, 100 μg/mL G-ADSC-Exos was significantly more effective compared with ≤ 100 μg/mL ADSC-Exos and < 100 μg/mL G-ADSC-Exos (*P* < 0.05), which indicated the better enhancing effect of G-ADSC-Exos than ADSC-Exos on the proliferation of the HUVECs under high-glucose conditions, and the most effective therapeutic concentration of G-ADSC-Exos was 100 μg/mL (Fig. 3B). Hence, the concentration of 100 μg/mL was adopted in the following experiments for G-ADSC-Exos and ADSC-Exos cocultured with the HUVECs. As for apoptosis assay, the Western blot demonstrated that the HUVECs cocultured with the ADSC-Exos and G-ADSC-Exos expressed a significantly lower level of Caspase-3 than the PBS group (*P* < 0.05), but the difference was not significant in the ADSC-Exo and G-ADSC-Exo groups. The G-ADSC-Exo group showed a significantly higher Bcl-2/Bax ratio than both the ADSC-Exo and control groups (*P* < 0.01), indicating the better anti-apoptotic effect of the G-ADSC-Exos over the ADSC-Exos on the HUVECs (Fig. 3C, D). The flow cytometry analysis indicated that the G-ADSC-Exo group possessed a lower percentage of early apoptosis (1.0 ± 0.2%) than the both ADSC-Exo (2.1 ± 0.4%) and control groups (8.2 ± 1.4%), which were significantly different (*P* < 0.05) (Fig. 3E, F). The above results proved that the G-ADSC-Exos improved the viability and inhibited the apoptosis of the HUVECs under high-glucose conditions.

### The G-ADSC-Exos improved the migration and tube formation of the HUVECs under high-glucose conditions

**Fig. 4:**
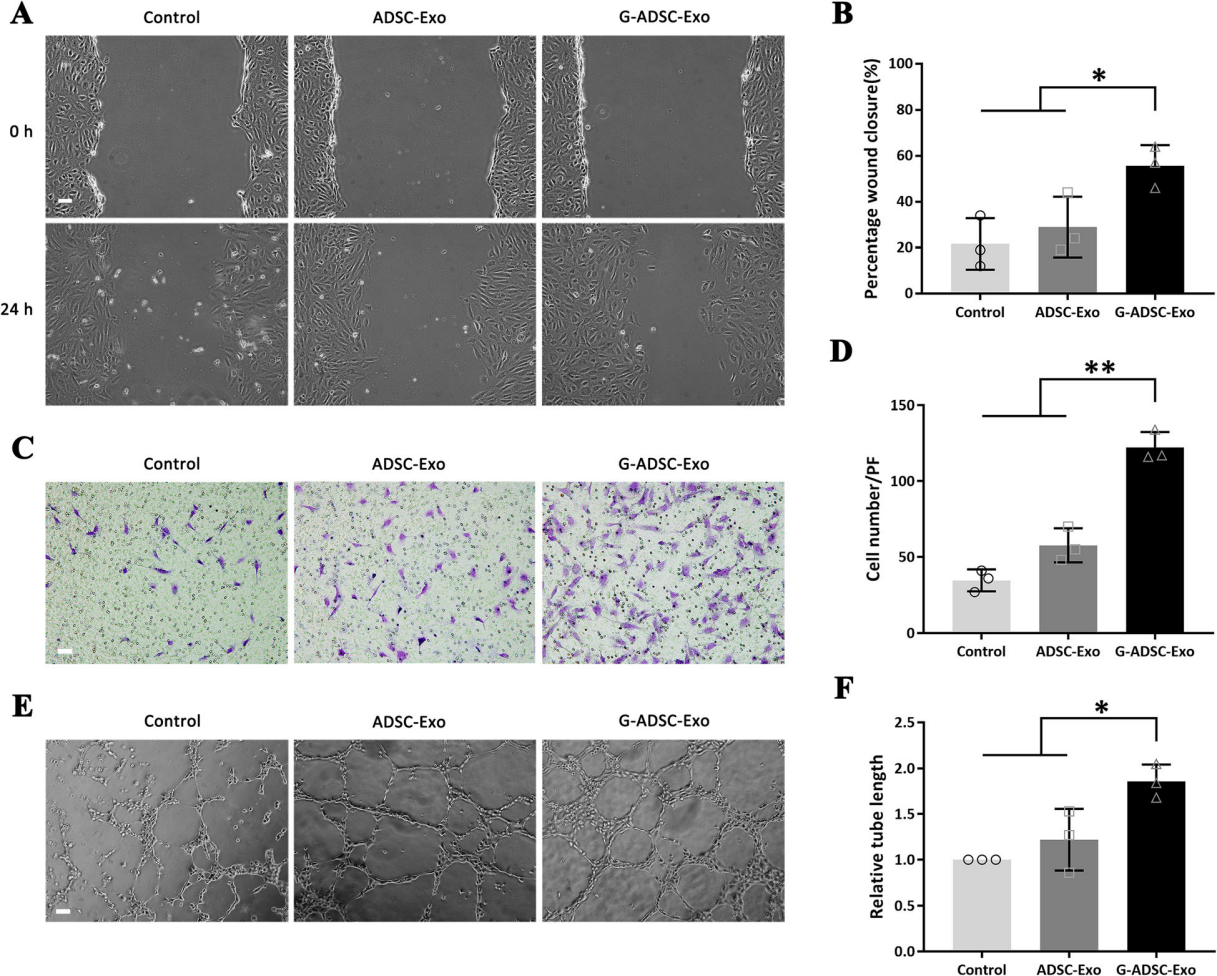
Wound-healing, transwell, and tube formation assay of the HUVECs. The wound-healing assay found a significantly higher wound closure percentage in the G-ADSC-Exo-treated HUVECs than the ADSC-Exo group and PBS group at 24 h under high-glucose conditions (**A**, **B**). The transwell assay showed a significantly larger number of migrated HUVECs in the G-ADSC-Exo group than the ADSC-Exo group and control group (**C**, **D**). The tube formation assay showed a significantly higher cumulative tubular length of the HUVECs cocultured with the G-ADSC-Exos than the ADSC-Exo and PBS groups (**E**, **F**). All experiments above were repeated three times (*n* = 3). The one-way analysis of variance was performed to compare data between groups. Scale bar = 100 μm; *P < 0.05; **P < 0.01. G-ADSC-Exos, G-ADSC-derived exosomes

The wound-healing assay revealed a significantly higher wound closure percentage in the G-ADSC-Exo-treated HUVECs (56 ± 9%) than the ADSC-Exo group (29 ± 13%) and the PBS group (22 ± 11%) at 24 h under high-glucose conditions (*P* < 0.05) (Fig. 4A, B). While the transwell assay revealed that the number of the migrated HUVECs was significantly larger in the G-ADSC-Exo group (122 ± 10 cells/field) than the both ADSC-Exo group (58 ± 11 cells/field) and control group (35 ± 7 cells/field) (*P* < 0.01) (Fig. 4C, D). Pertinent to tube formation assay, the HUVECs cocultured with the G-ADSC-Exos exhibited significantly higher cumulative tubular length than the ADSC-Exo and PBS groups (G-ADSC-Exo, 1.9 ± 0.2-folds; ADSC-Exo, 1.2 ± 0.3-folds of the PBS group, *P* < 0.05) (Fig. 4E, F). The results above verified that the G-ADSC-Exos significantly improved the migration and angiogenic ability of the HUVECs under high-glucose conditions.

### The G-ADSC-Exos enhanced the angiogenesis and blood perfusion and protected the muscle in the T2DM mouse limb ischemia model

**Fig. 5:**
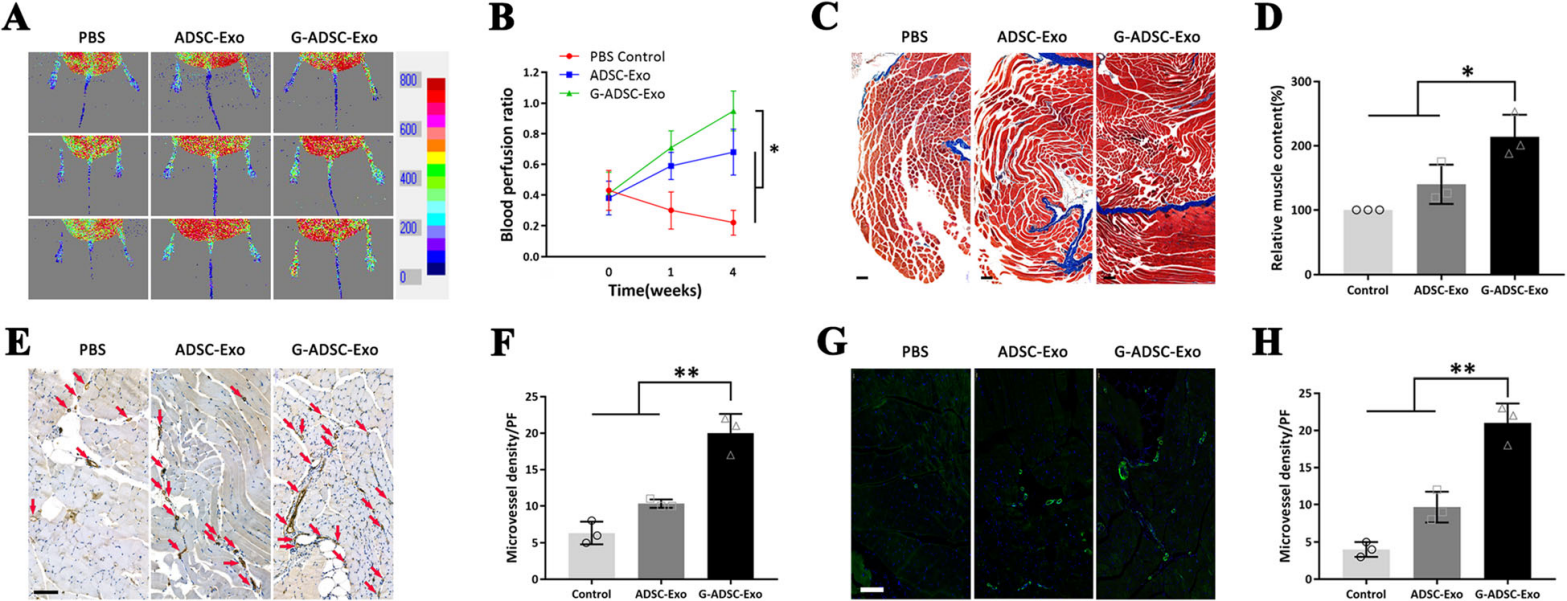
Blood perfusion and histological analysis of the T2DM mouse limb ischemia model after the G-ADSC-Exo transplantation. The blood flow of hindlimbs was monitored by the laser Doppler perfusion imager on days 0, 7, and 28 (**A**). Statistical analysis revealed a significantly higher blood perfusion ratio of the G-ADSC-Exo group than both ADSC-Exo and PBS control groups (**B**). The Masson’s staining indicated a significantly higher structural integrity of the ischemic muscles in the G-ADSC-Exo group than both ADSC-Exo and PBS control groups (**C**, **D**). The immunohistochemistry staining of CD31 in the hindlimb muscles showed a significantly higher microvessel’s density in the G-ADSC-Exo group than the ADSC-Exo and control groups (**E**, **F**). Immunofluorescence staining of α-SMA (green) and DAPI (blue) verified a significantly higher microvessel’s density in the G-ADSC-Exo group than the other two groups (**G**, **H**). All experiments above were repeated three times (*n* = 3). The one-way analysis of variance was performed to compare data between groups. Scale bar = 100 μm; *P < 0.05; **P < 0.01; ***P < 0.001; G-ADSC-Exos, G-ADSC-derived exosomes

In this animal experiment, a T2DM mouse model of simple low ligation of the femoral artery was constructed to primarily mimic the clinical peripheral vascular disease in T2DM patients, which has the process of vascular bed remodeling, progressive capillary loss, impaired neovascularization, and deteriorated ischemia [25, 26]. After transplantation of the exosomes, the blood flow of the hindlimbs was assessed and imaged by the laser Doppler perfusion imager on days 0, 7, and 28 (Fig. 5A). The captured images showed a significantly higher blood perfusion ratio of the G-ADSC-Exo group than the both ADSC-Exo and PBS control groups (G-ADSC-Exo group, 0.95 ± 0.13; ADSC-Exo group, 0.68 ± 0.15; PBS control group, 0.22 ± 0.08; *P* < 0.05) on the day 28 (Fig. 5B). The Masson’s trichrome staining and statistical analysis indicated a significantly higher structural integrity of the ischemic muscles in the G-ADSC-Exo group than the both ADSC-Exo and PBS control groups (relative muscle content: G-ADSC-Exo group, 2.1 ± 0.3-folds; ADSC-Exo group, 1.4 ± 0.3-folds as compared with the control group; *P* < 0.05) (Fig. 5C, D). And the immunohistochemistry staining of CD31 in the hindlimb muscles showed a significantly higher microvessel’s density in the G-ADSC-Exo group than the ADSC-Exo and control group (G-ADSC-Exo group, 20 ± 3 capillaries/field; ADSC-Exo group, 10 ± 1 capillaries/field; PBS control group, 6 ± 2 capillaries/field; *P* < 0.01) (Fig. 5E, F). The immunofluorescence staining of α-SMA and nucleus staining by DAPI, and statistical analysis further verified that the G-ADSC-Exo group possessed significantly higher microvessel’s density than the other two groups (G-ADSC-Exo group, 21 ± 3 capillaries/field; ADSC-Exo group, 10 ± 2 capillaries/field; PBS control group, 4 ± 1 capillaries/field; *P* < 0.01) (Fig. 5G, H). The above in vivo results indicated that the G-ADSC-Exos augmented the angiogenesis and blood perfusion and maintained the structural integrity of muscle in the T2DM mouse limb ischemia model.

### GLO-1 increased the paracrine factors in the ADSC-Exos

**Fig. 6:**
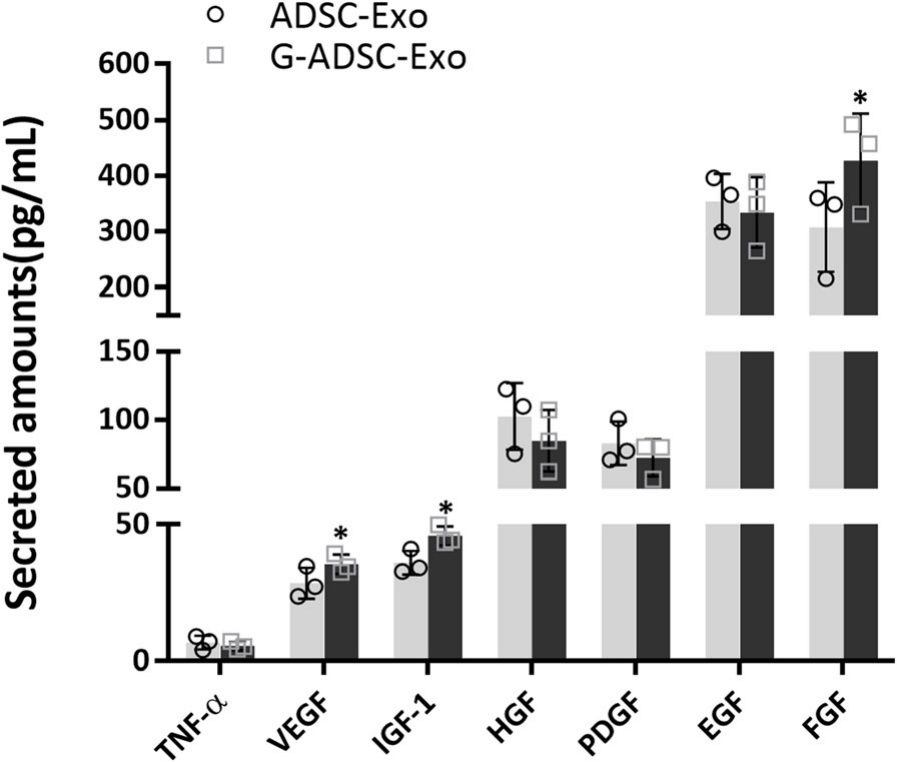
Detection of paracrine factors in the G-ADSC-Exos. The ELISA showed that the expression of VEGF, IGF-1, and FGF were significantly upregulated in the G-ADSC-Exos than the ADSC-Exos, but the differences in the expression of TNF-α, HGF, PDGF, and EGF were not significant between the two groups. All experiments above were repeated three times (*n* = 3). A two-tailed Student’s *t*-test was performed to compare data between groups. *P < 0.05; G-ADSC-Exos, G-ADSC-derived exosomes

The ELISA analysis of paracrine factors in the G-ADSC-Exos and ADSC-Exos demonstrated that the expression of VEGF, IGF-1, and FGF were upregulated in the G-ADSC-Exos than in the ADSC-Exos, and the differences were statistically significant (all *P* < 0.05). However, differences in the expression of TNF-α, HGF, PDGF, and EGF were not significant between the two groups (Fig. 6). The above result suggested that the G-ADSC-Exos contained more paracrine factors which might benefit the biological functions of the HUVECs.

### The regulation of the G-ADSC-Exos on the signaling pathways and ROS level in the HUVECs in vitro and in vivo

**Fig. 7:**
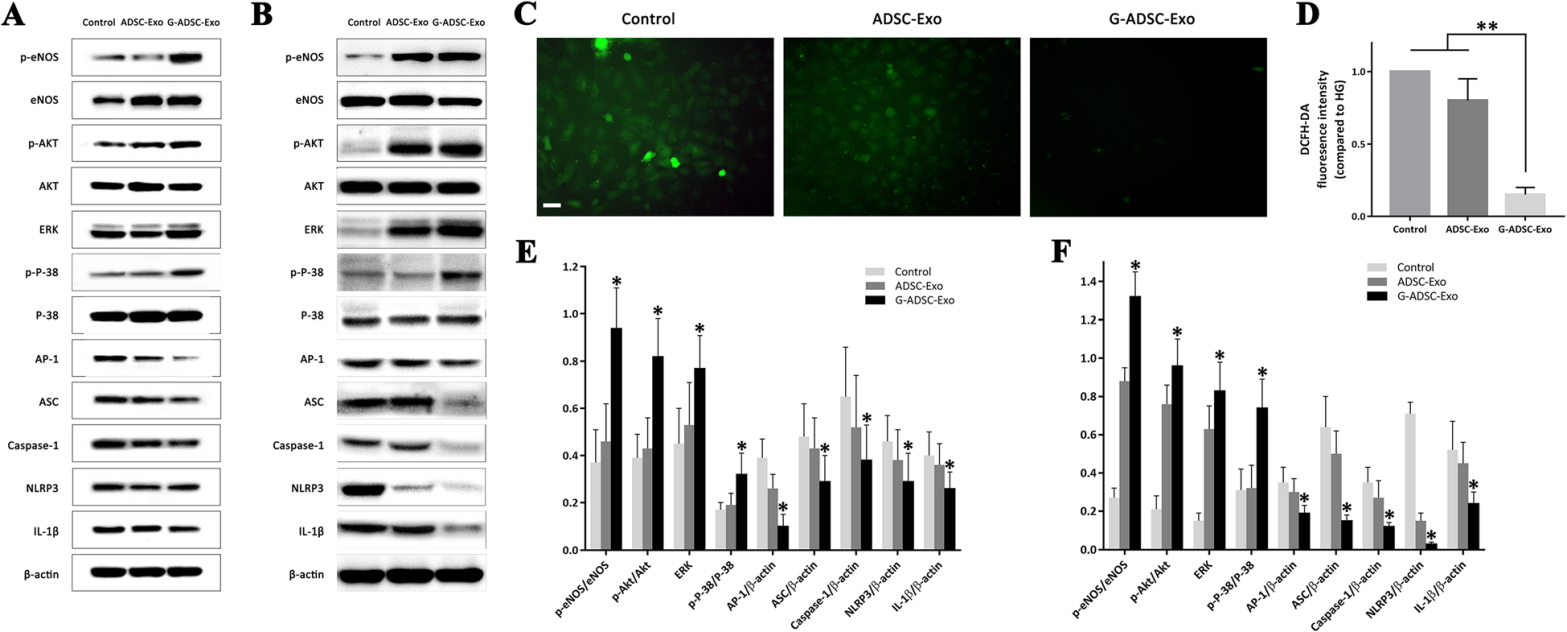
Detection of signaling pathways in vitro and in vivo and the ROS production in the HUVECs. Western blot indicated an elevation in the phosphorylation of eNOS/AKT/ERK/P-38 in the G-ADSC-Exo group than the ADSC-Exo and PBS group in vitro and in vivo, while the G-ADSC-Exo group showed significantly lower expression of AP-1, ASC, Caspase-1, NLRP3, and IL-1β than the ADSC-Exo and control group both in vitro and in vivo (**A**, **B**, **E**, **F**). The G-ADSC-Exo-treated HUVECs demonstrated a significantly lower fluorescence intensity than both ADSC-Exo and control groups under high-glucose conditions (**C**, **D**). All experiments above were repeated three times (*n* = 3). The one-way analysis of variance was performed to compare data between groups. *P < 0.05; **P < 0.01; scale bar = 50 μm; G-ADSC-Exos, G-ADSC-derived exosomes

Several previous studies demonstrated that the GLO-1 was the key rate-limiting enzyme in the glyoxalase system which reduced the accumulation of ROS in endothelial cells, and the ROS-NLRP3 pathway was proved to mediate the inflammation of endothelial cells under high-glucose conditions, leading to apoptosis and bio-functional disorders of the endothelial cells [27–29]. Hence, we focused on the ROS-NLRP3-related signaling molecules such as ASC (apoptosis-associated speck-like protein containing a CRAD), Caspase-1, and IL-1β as well as the classical signaling molecules such as eNOS, AKT, ERK, P-38, and AP-1. The Western blot and statistical analysis found an elevation in the phosphorylation of eNOS/AKT/ERK/P-38 in the G-ADSC-Exo group than the ADSC-Exo and PBS group in vitro and in vivo (*P* < 0.05), while the G-ADSC-Exo group showed significantly lower expression of AP-1, ASC, Caspase-1, NLRP3, and IL-1β than the ADSC-Exo and control groups both in vitro and in vivo (all *P* < 0.05) (Fig. 7A, B, E, F). As for the ROS level detected by a fluorescence microscope, the G-ADSC-Exo-treated HUVECs showed a significantly lower fluorescence intensity than both ADSC-Exo and PBS control groups under high-glucose conditions (*P* < 0.01) (Fig. 7C, D), which indicated a lower expression of ROS in the HUVECs. The activation of the eNOS/AKT/ERK/P-38 signaling pathways has been proved essential to the proliferation and migration of the HUVECs [30], while the downregulation of the AP-1, ASC, Caspase-1, NLRP3, IL-1β, and ROS might reduce the inflammation in the HUVECs induced by the high-glucose environment, which may also explain the better angiogenic ability of the HUVECs treated with G-ADSC-Exos under high-glucose conditions.

## Discussion

In the current study, the G-ADSC-Exos was efficiently isolated and identified, and the HUVECs cocultured with the G-ADSC-Exos exhibited significantly improved proliferation, migration, and tube formation and anti-apoptotic abilities in vitro in a high-glucose environment. A T2DM mouse model of simple low ligation of the femoral artery was constructed and in vivo transplantation of the G-ADSC-Exos significantly improved the blood perfusion, muscle structural integrity, and microvessel density in the ischemic hindlimbs. A preliminary study on the probable mechanisms revealed that the G-ADSC-Exos upregulated the eNOS/AKT/ERK/P-38 signaling, downregulated the AP-1/ROS/NLRP3/ASC/Caspase-1/IL-1β signaling pathway in the endothelial cells, and increased the secretion of VEGF, IGF-1, and FGF, which might explain the enhanced therapeutic effect of the G-ADSC-Exos on endothelial cells in a high-glucose environment.

This study, in general, was a continuation of our previous experiment which proved that the GLO-1 overexpression effectively protected the ADSCs against the ROS and improved the ADSC-mediated neovascularization in the diabetic hindlimb ischemia, but the low survival rate of the G-ADSCs after transplantation and use of the lentiviral transfection method made it an inefficient and unethical approach to treat diabetic hindlimb ischemia. Recently, researchers have been developing exosomes as stable natural nanocarriers to precisely deliver molecules and drugs to the targeted cells or tissues [31]. The specific development approaches of the exosomes included surface engineering, genetic engineering, chemical modification, and membrane fusion [32–34]. Of which the genetic engineering generated overexpression of a specific protein or microRNA and exhibited satisfactory outcome: Chen et al. found that the GDNF-modified human ADSCs-derived exosomes ameliorated peritubular capillary loss in tubulointerstitial fibrosis [35], Sun et al. revealed that the exosomes derived from the HIF-1α overexpressed mesenchymal stem cells (MSCs) enhanced the angiogenesis to provide cardioprotection in myocardial infarction [36], and Wang et al. discovered that exosomes derived from miR-155-5p-overexpressing synovial MSCs enhanced the bio functions of chondrocytes to prevent osteoarthritis [37]. In our preliminary experiment, we isolated the G-ADSC-Exos and demonstrated the high expression of GLO-1 protein in the G-ADSC-Exos. The ADSC-Exos was successfully genetically engineered to achieve efficient targeted delivery of GLO-1.

In diabetic lower limb ischemia, the tissue microenvironment possesses the characteristic of both ischemia and persistent hyperglycemia, which lead to the extensive accumulation of ROS, exacerbating oxidative stress and increasing inflammation in endothelial cells [38]. Recent studies showed that the NLRP3 (NACHT, LRR, and PYD domains-containing protein 3) inflammasome is a cytoplasmic protein complex mainly composed of the receptor protein, ASC, and pro-caspase-1. The pro-caspase-1 cleaves itself to produce active caspase-1, which then cleaves to form the pro-IL-1β and pro-IL-18, and releases their mature forms, mediating inflammation. The NLRP3 inflammasome has been reported as a key protein oligomer that causes endothelial cell inflammation and injury. The intracellular ROS can dissociate into thioredoxin-binding protein 2 (TXNIP, Thioredoxin-interacting protein) and thioredoxin (TRX, Thioredoxin). The TXNIP activates the NLRP3 inflammasome, and thus the ROS-TXNIP-NLRP3 pathway mediates inflammation and causes endothelial dysfunction [28, 29, 39]. The ROS acts as a common signal to activate the NLRP3 inflammasomes. The ROS inhibitors or scavengers inhibit the function of the NLRP3 inflammasomes, and the mitochondrial-derived ROS is pivotal in the activation of NLRP3 inflammasomes [40]. The GLO-1, as the key rate-limiting enzyme in the glyoxalase system, has been proved to effectively reduce the accumulation of ROS and improve the angiogenesis of endothelial cells [17–19]. In this study, we uncovered the downregulation of ASC, Caspase-1, NLRP3, IL-1β, and ROS as well as an inflammation-related transcription factor AP-1 in the G-ADSC-Exo group in vitro and in vivo, which indicated a reduction of inflammation in the endothelial cells. Moreover, the upregulation of classical eNOS/AKT/ERK/P-38 pathways positively regulated the proliferation and migration of the HUVECs. Taken together, we inferred that the above alterations in the signaling pathways were accounted for the improvement in proliferation, migration, and angiogenic ability of endothelial cells treated by G-ADSC-Exos.

However, the current study also had some limitations. First, although the T2DM mouse model of simple low ligation of the femoral artery has been reported to best mimic the diabetic lower limb ischemia of clinical patients [41], there still exist some differences in pathophysiological changes. The low ligation of the mouse femoral artery usually causes an acute occlusion of the trunk artery, whereas the clinical diabetic lower limb ischemia often begins with chronic occlusions of small arteries. Hence, further efforts should be made to perfect the mouse model mimicking the exact pathophysiological changes of a clinical diabetic lower limb ischemia. Second, the current study only performed a preliminary exploration of the underlying mechanisms by which the G-ADSC-Exos mediates the physiological functions of endothelial cells in a high-glucose environment. In this study, we only identified the cytokines such as VEGF, IGF-1, and FGF besides the GLO-1, ROS, NLRP3, and eNOS/AKT/ERK/P-38 pathways, and further study on the specific underlying mechanisms including the downstream signaling pathways of VEGF, IGF-1, and FGF are needed. Lastly, microRNAs have been reported to be pivotal in the contents of exosomes [42] and further elucidation on microRNAs is required in the future. Studies on miRNA sequencing from the G-ADSC-Exos have already been initiated by our research group, which will help better understand the composition and functions of the G-ADSC-Exos.

## Conclusion

This study found the crucial role of the G-ADSC-Exos in enhancing the viability, migration, and tube formation and possessing the anti-apoptotic property of the HUVECs in high-glucose in vitro environment. The in vivo transplantation of the G-ADSC-Exos promoted the angiogenesis and blood perfusion and protected the muscle in the T2DM mouse limb ischemia model, and the probable underlying mechanisms were the activation of eNOS/AKT/ERK/P-38 signaling pathways, inhibition of AP-1/ROS/NLRP3/ASC/Caspase-1/IL-1β, as well as increased secretion of VEGF, IGF-1, and FGF. Hence, the G-ADSC-Exos may be a promising therapeutic option for diabetic patients suffering from lower limb ischemia.

## Supplementary Information

**Additional file 1:** **Figure S1.** The blank control for ADSC surface markers with flow cytometry analysis. A supplement to Fig. 1A. **Figure S2.** The blank control, 7-AAD(+) control and PE(+) control for the apoptosis analysis of HUVECs treated with exosomes with flow cytometry. A supplement to Fig. 3E.

## Data Availability

Please contact the corresponding author for data requests.

## Abbreviations

ADSCs: Adipose-derived stem cells
GLO-1: Glyoxalase-1
*GLO-1*: GLO-1 gene
G-ADSCs: GLO-1-transfected ADSCs
G-ADSC-Exos: G-ADSC-derived exosome
HUVECs: Human umbilical vein endothelial cells
T2DM: Type 2 diabetes mellitus
DM: Diabetes mellitus
WT: Wild-type
DMEM: Dulbecco’s modified Eagle’s medium
FBS: Fetal bovine serum
PBS: Phosphate-buffered saline
GFP: Green fluorescent protein
MOI: Multiplicity of infection
NTA: Nanoparticle tracking analysis
TEM: Transmission electron microscope
CCK-8: Cell counting kit-8
7-AAD: 7-Amino-actinomycin D
PE: Phycoerythrin
PET: Polyethylene terephthalate
α-SMA: Alpha-smooth muscle actin
FITC: Fluorescein isothiocyanate
DAPI: 2-(4-Amidinophenyl)-6-indolecarbamidine dihydrochloride
TNF-α: Tumor necrosis factor-alpha
VEGF: Vascular endothelial growth factor
IGF-1: Insulin-like growth factor-1
HGF: Hepatocyte growth factor
PDGF: Platelet-derived growth factor
EGF: Epidermal growth factor
FGF: Fibroblast growth factor
DCFH-DA: Dichlorofluorescein diacetate
ROS: Reactive oxygen species
CM-Dil: Chloromethylbenzamino derivatives of 1,1′-dioctadecyl-3,3,3′,3′-tetramethylindocarbocyanine perchlorate
MSC: Mesenchymal stem cell
NLRP3: NACHT, LRR and PYD domains-containing protein 3
IL-1β: Interleukin-1β
TXNIP: Thioredoxin-interacting protein
TRX: Thioredoxin
ASC: Apoptosis-associated speck-like protein containing a CRAD
OD: Optical density

## References

[1] Nativel M, Potier L, Alexandre L, Baillet-Blanco L, Ducasse E, Velho G, Marre M, Roussel R, Rigalleau V, Mohammedi K (2018). Lower extremity arterial disease in patients with diabetes: a contemporary narrative review. Cardiovasc Diabetol.

[2] Jeffcoate W, Barron E, Lomas J, Valabhji J, Young B (2017). Using data to tackle the burden of amputation in diabetes. Lancet.

[3] Walsh JW, Hoffstad OJ, Sullivan MO, Margolis DJ (2016). Association of diabetic foot ulcer and death in a population-based cohort from the United Kingdom. Diabet Med.

[4] Brennan MB, Hess TM, Bartle B, Cooper JM, Kang J, Huang ES (2017). Diabetic foot ulcer severity predicts mortality among veterans with type 2 diabetes. J Diabetes Complications.

[5] Joret MO, Dean A, Cao C, Stewart J, Bhamidipaty V (2016). The financial burden of surgical and endovascular treatment of diabetic foot wounds. J Vasc Surg.

[6] Zhao L, Johnson T, Liu D (2017). Therapeutic angiogenesis of adipose-derived stem cells for ischemic diseases. Stem Cell Res Ther.

[7] Shi R, Jin Y, Cao C, Han S, Shao X, Meng L, Cheng J, Zhang M, Zheng J, Xu J, Li M (2016). Localization of human adipose-derived stem cells and their effect in repair of diabetic foot ulcers in rats. Stem Cell Res Ther.

[8] Dzhoyashvili NA, Efimenko AY, Kochegura TN, Kalinina NI, Koptelova NV, Sukhareva OY, Shestakova MV, Akchurin RS, Tkachuk VA, Parfyonova YV (2014). Disturbed angiogenic activity of adipose-derived stromal cells obtained from patients with coronary artery disease and diabetes mellitus type 2. J Transl Med.

[9] Kim H, Han JW, Lee JY, Choi YJ, Sohn YD, Song M, Yoon YS (2015). Diabetic mesenchymal stem cells are ineffective for improving limb ischemia due to their impaired angiogenic capability. Cell Transplant.

[10] Kim N, Cho SG (2015). New strategies for overcoming limitations of mesenchymal stem cell-based immune modulation. Int J Stem Cells.

[11] Adamiak M, Cheng G, Bobis-Wozowicz S, Zhao L, Kedracka-Krok S, Samanta A, Karnas E, Xuan YT, Skupien-Rabian B, Chen X, Jankowska U, Girgis M, Sekula M, Davani A, Lasota S, Vincent RJ, Sarna M, Newell KL, Wang OL, Dudley N, Madeja Z, Dawn B, Zuba-Surma EK (2018). Induced pluripotent stem cell (iPSC)-derived extracellular vesicles are safer and more effective for cardiac repair than iPSCs. Circ Res.

[12] Cabral J, Ryan AE, Griffin MD, Ritter T (2018). Extracellular vesicles as modulators of wound healing. Adv Drug Deliv Rev.

[13] Kalluri R, LeBleu VS (2020). The biology function and biomedical applications of exosomes. Science.

[14] Figliolini F, Ranghino A, Grange C, Cedrino M, Tapparo M, Cavallari C, Rossi A, Togliatto G, Femminò S, Gugliuzza MV, Camussi G, Brizzi MF (2020). Extracellular vesicles from adipose stem cells prevent muscle damage and inflammation in a mouse model of hind limb ischemia: role of Neuregulin-1. Arterioscler Thromb Vasc Biol.

[15] Groener JB, Oikonomou D, Cheko R, Kender Z, Zemva J, Kihm L, Muckenthaler M, Peters V, Fleming T, Kopf S, Nawroth PP (2019). Methylglyoxal and advanced glycation end products in patients with diabetes - what we know so far and the missing links. Exp Clin Endocrinol Diabetes.

[16] Yang P, Feng J, Peng Q, Liu X, Fan Z (2019). Advanced glycation end products: potential mechanism and therapeutic target in cardiovascular complications under diabetes. Oxid Med Cell Longev.

[17] Hanssen NM, Wouters K, Huijberts MS, Gijbels MJ, Sluimer JC, Scheijen JL (2014). Higher levels of advanced glycation endproducts in human carotid atherosclerotic plaques are associated with a rupture-prone phenotype. Eur Heart J.

[18] Vulesevic B, McNeill B, Geoffrion M, Kuraitis D, McBane JE, Lochhead M (2014). Glyoxalase-1 overexpression in bone marrow cells reverses defective neovascularization in STZ-induced diabetic mice. Cardiovasc Res.

[19] Zhiyou P, Xinrui Y, Jinbao Q, Kaichuang Y, Xin W, Huihua S (2017). Glyoxalase-1 overexpression reverses defective proangiogenic function of diabetic adipose-derived stem cells in streptozotocin-induced diabetic mice model of critical limb ischemia. Stem Cells Transl Med.

[20] Rothe M, Modlich U, Schambach A (2013). Biosafety challenges for use of lentiviral vectors in gene therapy. Curr Gene Ther.

[21] Xing Z, Jinbao Q, Xin W, Xin G, Junchao L, Xuhui W (2018). Netrin-1 improves adipose-derived stem cell proliferation, migration, and treatment effect in type 2 diabetic mice with sciatic denervation. Stem Cell Res Ther.

[22] Lobb RJ, Becker M, Wen SW, Wong CS, Wiegmans AP, Leimgruber A (2015). Optimized exosome isolation protocol for cell culture supernatant and human plasma. J Extracell Vesicles.

[23] Chen X, Duong MN, Psaltis PJ, Bursill CA, Nicholls SJ (2017). High-density lipoproteins attenuate high glucose-impaired endothelial cell signaling and functions: potential implications for improved vascular repair in diabetes. Cardiovasc Diabetol.

[24] Shen X, Zhang X, Ru W, Huang Y, Lan X, Lei C, Chen H (2020). circINSR Promotes proliferation and reduces apoptosis of embryonic myoblasts by sponging miR-34a. Mol Ther Nucleic Acids.

[25] Chen Y, Zhang J, Sun S (2014). Comparison of three approaches to establishing Balb/c mouse models of hind-limb ischemia. J South Med Univ.

[26] McDermott MM (2015). Lower extremity manifestations of peripheral artery disease: the pathophysiologic and functional implications of leg ischemia. Circ Res.

[27] Sachdeva R, Schlotterer A, Schumacher D, Matka C, Mathar I, Dietrich N, Medert R, Kriebs U, Lin J, Nawroth P, Birnbaumer L, Fleming T, Hammes HP, Freichel M (2018). TRPC proteins contribute to development of diabetic retinopathy and regulate glyoxalase 1 activity and methylglyoxal accumulation. Mol Metab.

[28] Abderrazak A, Syrovets T, Couchie D, El Hadri K, Friguet B, Simmet T (2015). NLRP3 inflammasome: from a danger signal sensor to a regulatory node of oxidative stress and inflammatory diseases. Redox Biol.

[29] Bai B, Yang Y, Wang Q, Li M, Tian C, Liu Y, Aung LHH, Li PF, Yu T, Chu XM (2020). NLRP3 inflammasome in endothelial dysfunction. Cell Death Dis.

[30] Na HJ, Hwang JY, Lee KS, Choi YK, Choe J, Kim JY, Moon HE, Kim KW, Koh GY, Lee H, Jeoung D, Won MH, Ha KS, Kwon YG, Kim YM (2014). TRAIL negatively regulates VEGF-induced angiogenesis via caspase-8-mediated enzymatic and non-enzymatic functions. Angiogenesis.

[31] Liang Y, Duan L, Lu J, Xia J (2021). Engineering exosomes for targeted drug delivery. Theranostics.

[32] Alvarez-Erviti L, Seow Y, Yin H, Betts C, Lakhal S, Wood MJ (2011). Delivery of siRNA to the mouse brain by systemic injection of targeted exosomes. Nat Biotechnol..

[33] Liang Y, Xu X, Li X, Xiong J, Li B, Duan L, Wang D, Xia J (2020). Chondrocyte-targeted microRNA delivery by engineered exosomes toward a cell-free osteoarthritis therapy. ACS Appl Mater Interfaces..

[34] Yang Y, Hong Y, Cho E, Kim GB, Kim IS (2018). Extracellular vesicles as a platform for membrane-associated therapeutic protein delivery. J Extracell Vesicles.

[35] Chen L, Wang Y, Li S, Zuo B, Zhang X, Wang F, Sun D (2020). Exosomes derived from GDNF-modified human adipose mesenchymal stem cells ameliorate peritubular capillary loss in tubulointerstitial fibrosis by activating the SIRT1/eNOS signaling pathway. Theranostics.

[36] Sun J, Shen H, Shao L, Teng X, Chen Y, Liu X, Yang Z, Shen Z (2020). HIF-1α overexpression in mesenchymal stem cell-derived exosomes mediates cardioprotection in myocardial infarction by enhanced angiogenesis. Stem Cell Res Ther.

[37] Wang Z, Yan K, Ge G, Zhang D, Bai J, Guo X, Zhou J, Xu T, Xu M, Long X, Hao Y, Geng D (2021). Exosomes derived from miR-155-5p-overexpressing synovial mesenchymal stem cells prevent osteoarthritis via enhancing proliferation and migration, attenuating apoptosis, and modulating extracellular matrix secretion in chondrocytes. Cell Biol Toxicol.

[38] Incalza MA, D'Oria R, Natalicchio A, Perrini S, Laviola L, Giorgino F (2018). Oxidative stress and reactive oxygen species in endothelial dysfunction associated with cardiovascular and metabolic diseases. Vascul Pharmacol.

[39] Lane T, Flam B, Lockey R, Kolliputi N (2013). TXNIP shuttling: missing link between oxidative stress and inflammasome activation. Front Physiol.

[40] Zhou R, Yazdi AS, Menu P, Tschopp J (2011). A role for mitochondria in NLRP3 inflammasome activation. Nature.

[41] Portou MJ, Yu R, Baker D, Xu S, Abraham D, Tsui J (2020). Hyperglycaemia and ischaemia impair wound healing via toll-like receptor 4 pathway activation in vitro and in an experimental murine model. Eur J Vasc Endovasc Surg.

[42] Asgarpour K, Shojaei Z, Amiri F, Ai J, Mahjoubin-Tehran M, Ghasemi F, ArefNezhad R, Hamblin MR, Mirzaei H (2020). Exosomal microRNAs derived from mesenchymal stem cells: cell-to-cell messages. Cell Commun Signal.

