# Supplementary material for: Exosomes derived from adipose-derived stem cells overexpressing glyoxalase-1 protect endothelial cells and enhance angiogenesis in type 2 diabetic mice with limb ischemia
Source: Stem Cell Res Ther. 2021 Jul 15;12:403. doi: 10.1186/s13287-021-02475-7 (PMC8281719; doi:10.1186/s13287-021-02475-7)
Supplement: Supplementary file 1 — Additional file 1: Figure S1. The blank control for ADSC surface markers with flow cytometry analysis. A supplement to Fig. 1A. Figure S2. The blank control, 7-AAD(+) control and PE(+) control for the apoptosis analysis of HUVECs treated with exosomes with flow cytometry. A supplement to Fig. 3E. [file 13287_2021_2475_MOESM1_ESM.docx]

**Supplementary materials**

**
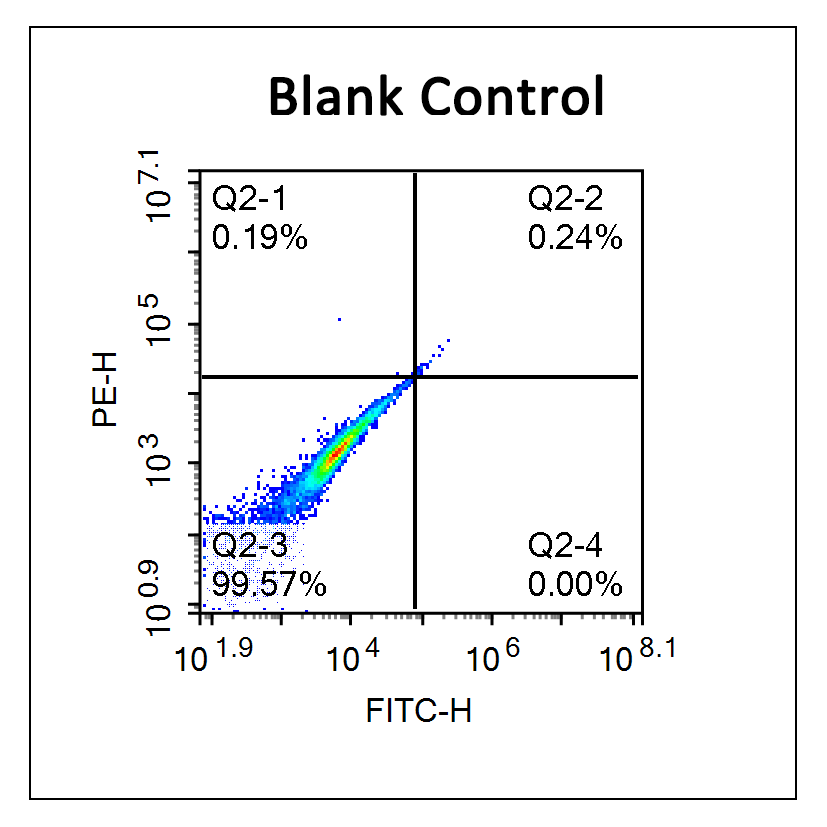
**

**Figure S1** The blank control for ADSC surface markers with flow cytometry analysis. A supplement to Figure 1A.


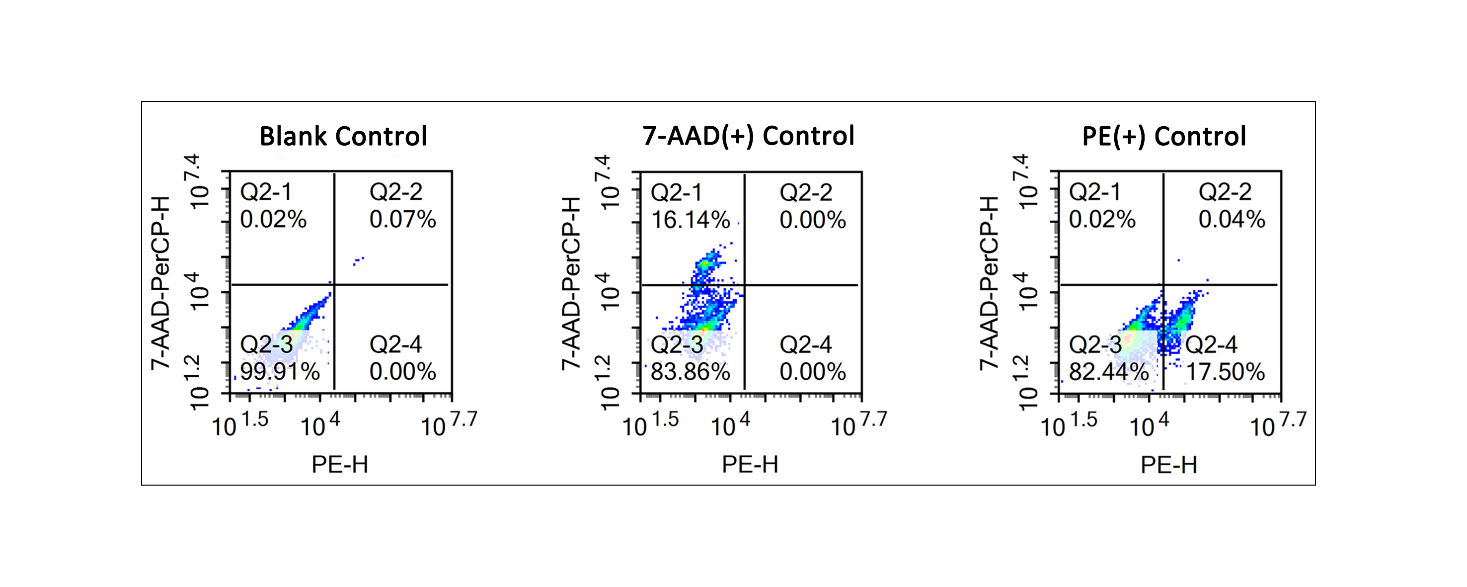


**Figure S2** The blank control, 7-AAD(+) control and PE(+) control for the apoptosis analysis of HUVECs treated with exosomes with flow cytometry. A supplement to Figure 3E.
